# Supplementary material for: Jointly exploring client drift and catastrophic forgetting in dynamic learning
Source: Sci Rep. 2025 Feb 18;15:5857. doi: 10.1038/s41598-025-89873-6 (PMC11836390; doi:10.1038/s41598-025-89873-6)
Supplement: Supplementary file 2 — Supplementary Information 2. [file 41598_2025_89873_MOESM2_ESM.pdf]

Supplementary Material: Jointly Exploring Client Drift and Catastrophic Forgetting in Dynamic Learning

Niklas Babendererde[1,a*], **Moritz Fuchs**[1,b], **Camila Gonzalez**[2,c], **Yuri Tolkach**[3,d], **and Anirban Mukhopadhyay**[1,e]

[1]TU Darmstadt, Computer Science, Darmstadt, Germany
[2]Stanford University, Computational Neuroscience Laboratory, Stanford, United States
[3]University Hospital Cologne, Institute of Pathology, Cologne, Germany
[a]niklas.babendererde@gris.tu-darmstadt.de
[b]moritz.fuchs@gris.informatik.tu-darmstadt.de
[c]camgonza@stanford.edu
[d]iurii.tolkach@uk-koeln.de
[e]anirban.mukhopadhyay@gris.tu-darmstadt.de

1 Transformations on CelebA

1.1 Ablation Study

In the following, we investigate how the given transformation types for CelebA impact CD and CF. We perform the usual experiments for each transformation type individually at full strength and in both scenarios but with fewer epochs due to the large computational effort. We train for **100 global epochs** in the CD scenario and **50 additional epochs** in the CF scenario. Figure 1 visualizes the resulting relative accuracy drop in CD and Figure 2 in CF compared to the shift-free baseline. It shows that the contrast transformation has the largest impact in both scenarios. As the goal is to classify if the person is smiling, a change in contrast, can reduce the visibility of the mouth in the face, which explains why this degrades the performance. Moreover, noise transformations have a large impact as they disturb the ability to detect the structure of the mouth.

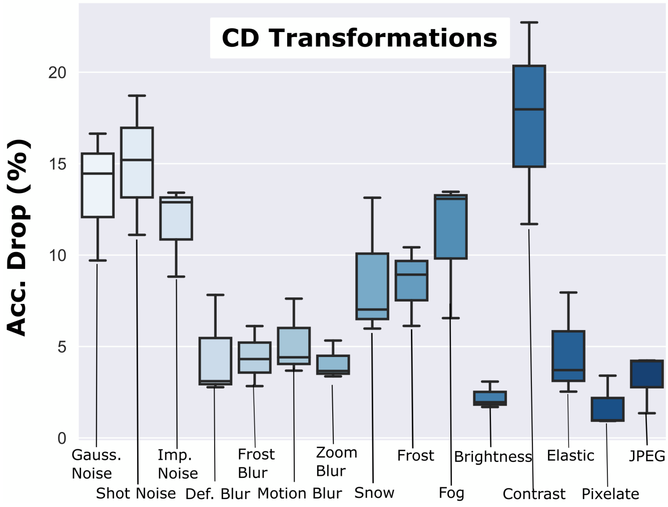

Figure 1. Relative performance drop from the specific transformations in the CD Experiment of CelebA

2 Transformations on PESO

2.1 Calibration

To ensure that the transformations are usable for a consistent emulation of distribution shifts, we perform the following calibration process.

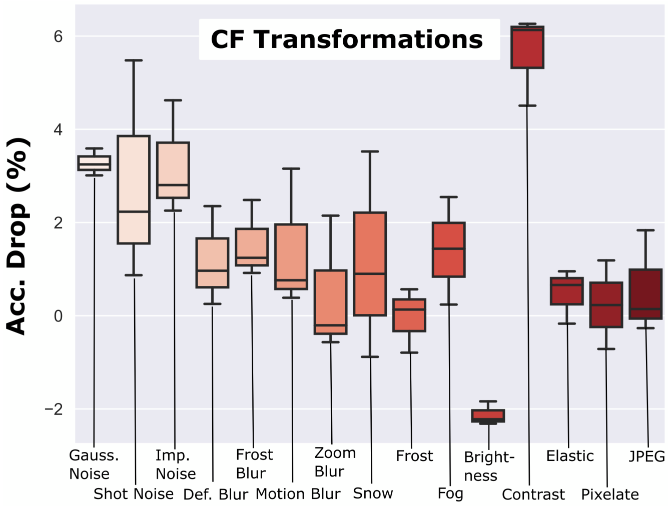

Figure 2. Relative performance drop from the specific transformations in the CF Experiment of CelebA

For the overlay-based transformations that add artifacts, such as the thread on top of the existing image, we set the transformation severity with the affected image area scaling to ensure that a certain share of the image is covered. Moreover, we configure the opacity to control what details remain visible under the overlay. In the case of the brightness transformation, we configure the severity by changing the brightness factor.

In the first calibration step, we tune these parameters in such a way that the Dice Score on our pretrained standard federation drops by 20% from 94% on clean test data to 75% with the respective transformation. After that, we perform two more tests to ensure that these transformations are feasible to emulate CF. We first apply the resulting transformation to train the same federation for the same 500 epochs required to achieve convergence. The final model is tested on clean data, and we confirmed that the Dice Score drops by at least 5% from this distribution shift. In the last step, we test for the ability to emulate CF. Therefore, we test the clean pretrained model separately on clean and transformed data. At the beginning of the distribution shift, we expect a higher Dice Score on clean data than on transformed data. In the following, we expect a changing order. If this is the case, this indicates CF. We perform all the stated tests with the transformations used in this work to ensure their feasibility for consistent distribution shifts. Figure 3 shows samples of the resulting transformations on PESO.

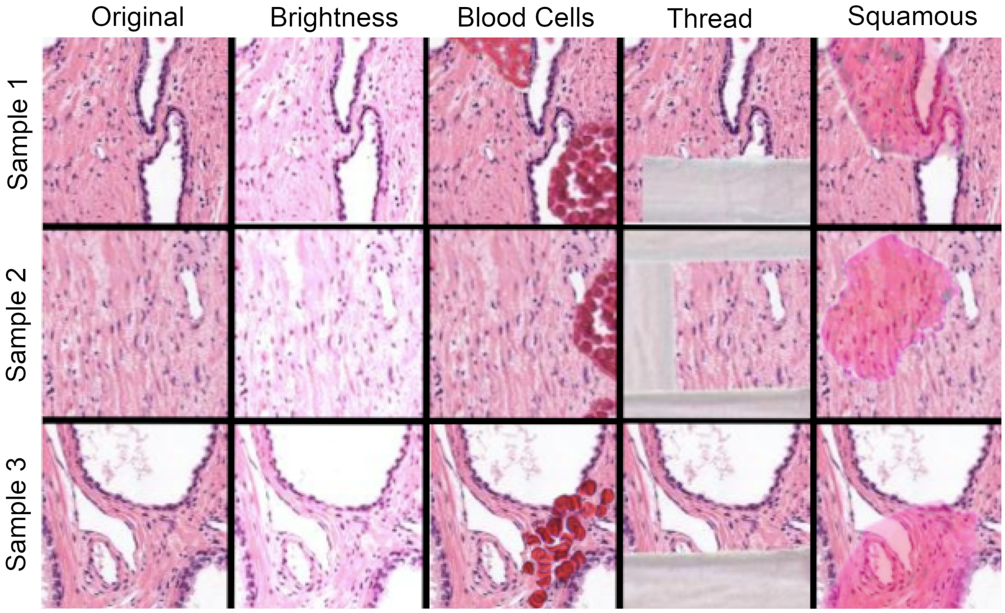

Figure 3. Samples of the transformations applied to the Whole Slide Images from the PESO dataset

Problem	Meth. Category	Method
Client Drift (CD)	Penalizing Outliers	*FedAvgM*[1]
		FedProx[2]
	Compensate	*Scaffold*[3]
	Knowledge Distillation (KD)	*Hinton et al.*[4]
		FedDF[5]
		FedMD[6]
		CRONUS[7]
		FedAUX[8]
	Data Sharing	*FedMatch*[9]
Catastrophic Forgetting (CF)	Param. Tuning	*Donahue et al.*[10]
		Sharif et al.[11]
		Girshick et al.[12]
	Network Extension	*Terekhov et al.*[13]
		Rusu et al.[14]
	Keep data/logits	*Multitask Learn.*[15]
		Rehearsal[16]
		Li et al.[17]

Table 1. Overview of the existing Federated and Continual Learning methods tackling, respectively, Client Drift and Catastrophic Forgetting.

3 Related Work

In Table 1 we give an overview of existing approaches that address CD/CF individually and categorize them. We start with a short introduction of the main categories from the table for approaches against CD: One direction is to either just *penalize outliers*[2] while other methods go further by *actively compensating CD*[3]. Alternatively, sharing a global dataset enables a better generalization and makes the model less prone to CD[9]. A very different approach is to apply *Knowledge Distillation*[4]: First, the clients train their local model. After that, they teach the server by providing their output logits. Besides making the global model more flexible to different inputs, this intermediate representation step reduces CD. Beyond these FL-related methods, there are separate approaches against CF in CL: One direction is *parameter tuning*[10] to balance learning new data distributions while preserving the existing knowledge. Alternatively, some approaches *extend the network*[14] to keep distribution-specific weights. *Keeping data/logits*[16] is a widely used approach, which we also apply in our experiments.

4 Rehearsal

Rehearsal[16] is a widely used method to handle CF in Continual Learning settings. It stores a small share of the data after each training stage and continuously interleaves old with new samples in future training epochs. We apply this concept to a federated setting: **Each client trains on an additional certain share of data that remains untouched by the shifting transformation**. Using only local data, **ensures that no private data of a client is leaked to the global model or any other client**. The share of additional clean data makes up 20% of the train data of each local client. Figure 4 illustrates the concept of rehearsal in a federated setting. We use our framework to test rehearsal in scenarios of CD as well as CF compared to the baseline with all clients shifted in CD and at the strongest shift strength level in the CD-scenario.

Figure 5 shows the impact of rehearsal on the CD/CF Scenario. As expected from a method against CF, it decreases the performance drop on CelebA by **82.63%** from 1.24% to 0.12%. However, this commonly used CF-method also shows improvements in the CD Scenario, as it decreases the performance drop by **51.05%** from 3.45% on CelebA to only 1.69%. This motivates to jointly analyze both problems, as in Figure 9 of the main paper, which highlights the persistence of the **generalization bump**.

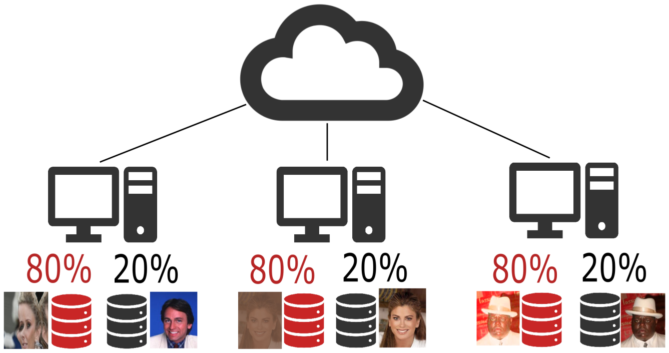

Figure 4. Rehearsal in FL: The clients mix their potentially shifted local train data with 20% ID data for improvements in scenarios of CD and CF

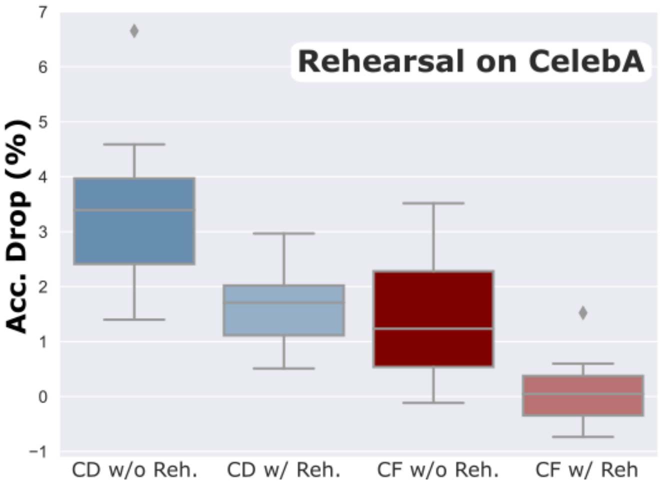

Figure 5. Performance impact from rehearsal on CD/CF

5 Experiment Details

5.1 Parameter and Environment

To improve reproducibility and make the setup more comprehensible, Table 2 lists relevant parameters and details regarding the environment. It extends the written description from the main paper with additional details.

5.2 Algorithms

Algorithm 1 sketches the application of the joint CD/CF experiment as a pseudocode. $max_{strength}$ represents the maximum shift strength that the corresponding transformation can cause. In case of CelebA this means $max_{strength} = 5$ with a step size of 1 due to the fixed shift strength levels. For PESO, we assume $max_{strength} = 1$ with an arbitrarily small step size due to its continuous scale. To make the pseudocode more comprehensive, we don't include a possible efficiency improvement to the pseudocode: Skipping the initialization round for CD instead after the first CD-round would save many training epochs. However, this has no impact on the results and should be easier to understand.

References

1. Hsu, H., Qi, H. & Brown, M. Measuring the effects of non-identical data distribution for federated visual classification. *arXiv preprint arXiv:1909.06335* (2019).
2. Li, T. *et al.* Federated optimization in heterogeneous networks. *Proc. Mach. Learn. Syst.* **2**, 429–450 (2020).
3. Karimireddy, S. P. *et al.* Scaffold: Stochastic controlled averaging for federated learning. In *International Conference on Machine Learning*, 5132–5143 (PMLR, 2020).
4. Hinton, G., Vinyals, O. & Dean, J. Distilling the knowledge in a neural network. *stat* **1050**, 9 (2015).

	CelebA	PESO
Model	*ViT*[18]	*U-Net*[19]
Model Implementation	*ViT-FL*[20]	*BottleGAN*[21]
Federated Algorithm	*FedAvg*[22]	*FedAvg*[22]
Local Epochs	1	1
Global Epochs (CD)	200	500
Global Epochs after shift (CF)	100	40
Total Clients	227	10
Clients per Global Epoch	10	4
Seeds	[1,2,3,4,5,6,7,8,9,10]	[1,2,3]
Loss	Cross Entropy	Cross Entropy
Optimizer	SGD	Adam
Optimizer param.	-	$\beta_1 = 0.9$, $\beta_2 = 0.999$
Learning rate	0.02	0.001
Step Size	30	100
Batch Size	32	48
Image/Patch Size	178×218	114×114
GPU	NVIDIA GeForce RTX 4090	NVIDIA A40
CUDA SDK Version	12.2	11.6
PyTorch Version	2.0.0	1.13.0

Table 2. Parameters and Environment for the experiments on PESO

Algorithm 1 Joint Analysis of CD and CF

Input: *seeds*
Require: $shifts_{cd} = [0, 1]$
  and $shifts_{cf} = [0, max\_strength]$
  for *seed* in *seeds* **do**
    for $shift_{cd}$ in $shifts_{cd}$ **do**
      for $shift_{cf}$ in $shifts_{cf}$ **do**
        $model \leftarrow init(seed)$
        $perf_{cdcf} \leftarrow 0$
        for *epoch* in *cd_epochs* **do**
          $model \leftarrow train(model, shift_{cd}, seed, shift\_strength = 1.0)$
        end for
        if $shift_{cd} == 0$ **and** $shift_{cf} == 0$ **then**
          $perf_{id} \leftarrow metric(test(model, data_{id}))$
        end if
        for *epoch* in *cf_epochs* **do**
          $model \leftarrow train(model, shift\_ratio = 1.0, seed, shift_{strength} = shift_{cf})$
        end for
        $perf_{cdcf} \leftarrow metric(test(model, data_{id}))$
        $\blacksquare perf_{cdcf}(shift_{cd}, shift_{cf}) \leftarrow perf_{id} - perf_{cdcf}$
      end for
    end for
  end for
  return $\blacksquare perf_{cdcf}$

5. Lin, T., Kong, L., Stich, S. U. & Jaggi, M. Ensemble distillation for robust model fusion in federated learning. *Adv. Neural Inf. Process. Syst.* **33**, 2351–2363 (2020).

6. Li, D. & Wang, J. Fedmd: Heterogenous federated learning via model distillation. *arXiv preprint arXiv:1910.03581* (2019).

7. Chang, H., Shejwalkar, V., Shokri, R. & Houmansadr, A. Cronus: Robust and heterogeneous collaborative learning with black-box knowledge transfer. *arXiv preprint arXiv:1912.11279* (2019).

8. Sattler, F., Korjakow, T., Rischke, R. & Samek, W. Fedaux: Leveraging unlabeled auxiliary data in federated learning. *IEEE Transactions on Neural Networks Learn. Syst.* (2021).

9. Jeong, W., Yoon, J., Yang, E. & Hwang, S. J. Federated semi-supervised learning with inter-client consistency & disjoint learning. In *International Conference on Learning Representations (ICLR) 2021* (International Conference on Learning Representations (ICLR), 2021).

10. Donahue, J. *et al.* Decaf: A deep convolutional activation feature for generic visual recognition. In *International conference on machine learning*, 647–655 (PMLR, 2014).

11. Sharif Razavian, A., Azizpour, H., Sullivan, J. & Carlsson, S. Cnn features off-the-shelf: an astounding baseline for recognition. In *Proceedings of the IEEE conference on computer vision and pattern recognition workshops*, 806–813 (2014).

12. Girshick, R., Donahue, J., Darrell, T. & Malik, J. Rich feature hierarchies for accurate object detection and semantic segmentation. In *Proceedings of the IEEE conference on computer vision and pattern recognition*, 580–587 (2014).

13. Terekhov, A. V., Montone, G. & O'Regan, J. K. Knowledge transfer in deep block-modular neural networks. In *Conference on Biomimetic and Biohybrid Systems*, 268–279 (Springer, 2015).

14. Rusu, A. A. *et al.* Progressive neural networks. *arXiv preprint arXiv:1606.04671* (2016).

15. Caruana, R. Multitask learning. *Mach. learning* **28**, 41–75 (1997).

16. Rebuffi, S.-A., Kolesnikov, A., Sperl, G. & Lampert, C. H. icarl: Incremental classifier and representation learning. In *Proceedings of the IEEE conference on Computer Vision and Pattern Recognition*, 2001–2010 (2017).

17. Li, Z. & Hoiem, D. Learning without forgetting. *IEEE transactions on pattern analysis machine intelligence* **40**, 2935–2947 (2017).

18. Dosovitskiy, l. *et al.* An image is worth 16x16 words: Transformers for image recognition at scale. In *ICLR 2021*, 10061–10071 (2022).

19. Ronneberger, O., Fischer, P. & Brox, T. U-net: Convolutional networks for biomedical image segmentation. In *Medical Image Computing and Computer-Assisted Intervention–MICCAI 2015: 18th International Conference, Munich, Germany, October 5-9, 2015, Proceedings, Part III 18*, 234–241 (Springer, 2015).

20. Qu, L. *et al.* Rethinking architecture design for tackling data heterogeneity in federated learning. In *Proceedings of the IEEE/CVF Conference on Computer Vision and Pattern Recognition*, 10061–10071 (2022).

21. Wagner, N., Fuchs, M., Tolkach, Y. & Mukhopadhyay, A. Federated stain normalization for computational pathology. In *Medical Image Computing and Computer Assisted Intervention–MICCAI 2022: 25th International Conference, Singapore, September 18–22, 2022, Proceedings, Part II*, 14–23 (Springer, 2022).

22. McMahan, B., Moore, E., Ramage, D., Hampson, S. & Arcas, B. A. y. Communication-Efficient Learning of Deep Networks from Decentralized Data. In Singh, A. & Zhu, J. (eds.) *Proceedings of the 20th International Conference on Artificial Intelligence and Statistics*, vol. 54 of *Proceedings of Machine Learning Research*, 1273–1282 (PMLR, 2017).
